# Investigation and Development of the BODIPY-Embedded Isotopic Signature for Chemoproteomics Labeling and Targeted Profiling

**DOI:** 10.1021/jasms.4c00246

**Published:** 2024-09-16

**Authors:** Rachel Joshi, Adam M. Hawkridge

**Affiliations:** † Department of Medicinal Chemistry, Virginia Commonwealth University, Richmond, Virginia 23219, United States; ‡ Department of Pharmaceutics, Virginia Commonwealth University, Richmond, Virginia 23298-0533, United States

## Abstract

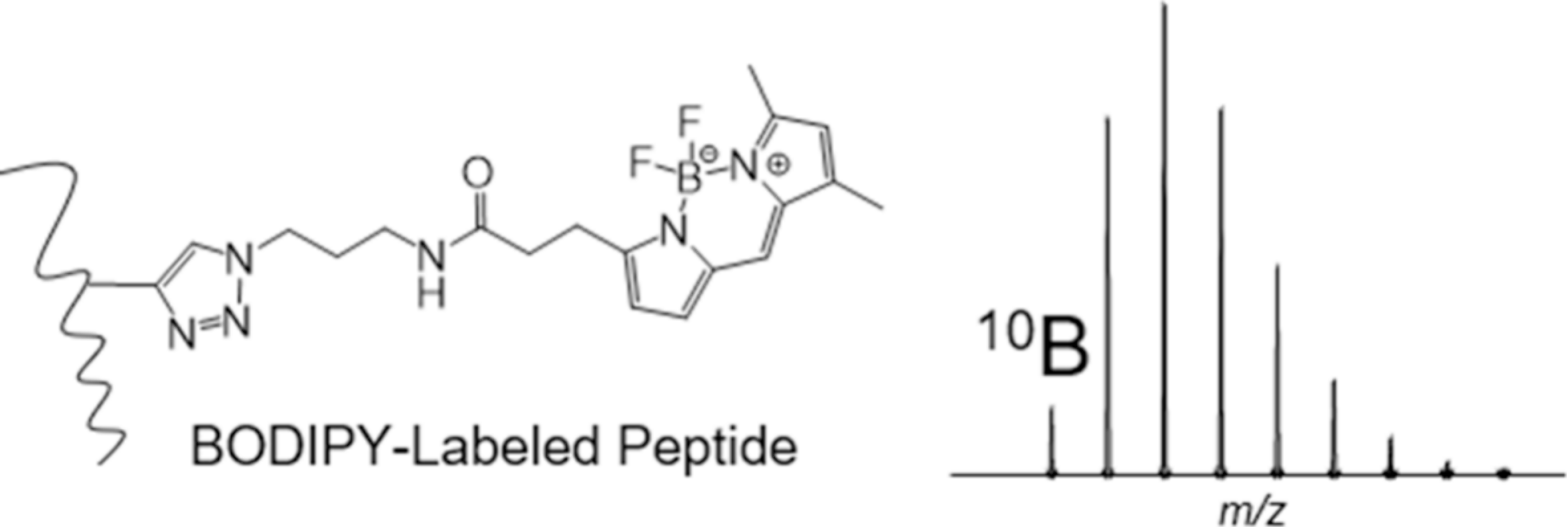

A common goal in mass spectrometry-based chemoproteomics is to directly measure the site of conjugation between the target protein and the small molecule ligand. However, these experiments are inherently challenging due to the low abundance of labeled proteins and the difficulty in identifying modification sites using standard proteomics software. Reporter tags that either generate signature fragment ions or isotopically encode target peptides can be used for the preemptive discovery of labeled peptides even in the absence of identification. We investigated the potential of BODIPY FL azide as a click chemistry enabled chemoproteomics reagent due to the presence of boron and the unique 1:4 natural abundance ratio of ^10^B:^11^B. The isotopes of boron encode BODIPY-labeled peptides with a predictable pattern between the monoisotopic (M) and M+1 peaks. BODIPY-labeled peptides were identified in MS1 spectra using an R script that filters for the signature ^10^B:^11^B intensity ratio and mass defect. Application of the boron detection script resulted in three times the labeled peptide coverage achieved for a BODIPY-conjugated BSA sample compared with untargeted data-dependent acquisition sequencing. Furthermore, we used the inherent HF neutral loss signature from BODIPY to assist with BODIPY-modified peptide identification. Finally, we demonstrate the application of this approach using the BODIPY-conjugated BSA sample spiked into a complex *E. coli*. digest. In summary, our results show that the commercially available BODIPY FL azide clicked to alkyne-labeled peptides provides a unique isotopic signature for pinpointing the site(s) of modification with the added potential for on- or off-line UV or fluorescence detection.

## Introduction

Mass spectrometry-based chemoproteomics such as photoaffinity labeling (PAL)^1^ and activity-based protein profiling (ABPP)^2^ have become powerful methods for drug discovery in cancer, diabetes and immunology.^3−7^ The most common goal in a chemoproteomics experiment is identifying protein(s) that specifically interact with the probe of interest (e.g., drug, fragment, etc.). The protein-bound probe typically contains a bio-orthogonal handle (e.g., alkyne, azide) that can be clicked to a bead or biotin for subsequent enrichment, enzymatic digestion, and LC-MS/MS analysis. A secondary more challenging goal is to identify the peptide-probe conjugate and thereby localizing the binding site.^8,9^ Advances in probe chemistry,^10−12^ tagging strategies^13−15^ and peptide sequencing software^16,17^ have facilitated efficient identification of peptides bearing covalent modifications to unknown amino acid residues. Despite these advances, technical challenges remain in terms of peptide-probe identification due to inherent low conversion efficiency of the photolabeling process, bias against low abundant precursor ions with data-dependent acquisition (DDA), and limitations of commercial software that often cannot cover sufficient search space to account for unknown or heterogeneous modifications.^18,19^

Reporter tags that generate diagnostic signature fragment ions via collision induced dissociation (CID) can be used to preemptively locate peptide-probe candidates even in the absence of identification. Therefore, the discovery of labeled peptides is independent from the sequencing software and blind to the photolabeling mechanism. Signature ions have been used broadly in proteomics to select for phosphorylation,^20^ glycosylation^21,22^ and other PTMs.^23−25^ Biotinylation, which is often employed in chemoproteomics to enrich labeled proteins from biological matrices, can be identified by multiple diagnostic oxonium signature ions.^26^ More recently signature ions were reported for the triazole ring that is specifically formed by copper-catalyzed azide–alkyne cycloaddition (CuAAC), the bioconjugate “click” reaction commonly used to functionalize labeled proteins.^27^

Alternatively, chemoproteomic-labeled peptides can be isotopically encoded using reporter tags that capitalize on the high natural abundance of the heavy isotopes of chlorine (^37^Cl, 24%) and bromine (^81^Br, 49%).^28−32^ The enhanced M+2 peak in the halogenated peptide isotopic distributions can be detected visually or using computational tools, such as the IsoStamp algorithm developed by Bertozzi and co-workers to detect peptides bearing a dibromide modification.^30^ In their work, eluting bovine serum albumin peptides were analyzed with continuous MS1 scans and processed through IsoStamp to filter for halogenated peptides, which were then subjected to targeted MS2 experiments. Targeted profiling resulted in a nearly 2-fold increase in labeled peptide coverage than was achieved with DDA in a Jurkat cell lysate. Similarly, Gao et al. developed an algorithm to computationally identify the selenium isotopes in endogenous selenoproteins and reported a 1 order of magnitude increase in sensitivity compared with DDA by spiking selenopeptides into a HeLa lysate.^33^ Lastly, Yang et al. expanded on the earlier IsoStamp studies using a dibromide coumarin azide tag^32^ that enabled the orthogonal detection of tagged peptides by in-line fluorescence spectroscopy prior to MS analysis.

Here we investigated the use of BODIPY (4,4-difluoro-4-bora-3a,4a-diaza-*s*-indacene) FL azide,^34^ a commercially available clickable dye used for fluorescence spectroscopy and imaging,^35,36^ as a new chemoproteomics labeling tag. BODIPY includes a single boron atom, which has two stable isotopes, ^10^B (20%) and ^11^B (80%),^37^ that produce a unique isotopic signature when coupled to complex organic molecules^38^ and peptides.^39^ Furthermore, BODIPY-modified peptides can be orthogonally detected by UV–vis and fluorescence spectroscopy. Three analytical figures of merit were assessed in this study to enable the identification of BODIPY-tagged versus untagged tryptic peptides: (1) the ^10^B monoisotopic peak abundance serves as a distinguishing isotopic feature, (2) ^10^B imparts a distinguishing mass defect between M and M+1 that can be resolved using high resolution accurate mass (HRAM) instruments, and (3) BODIPY produces a signature neutral loss of HF both in the electrospray source and during collision induced dissociation. Finally, we developed an automated script for detecting BODIPY-labeled peptides in complex proteomics mixtures. Collectively, this work introduces the novel use of a commercially available tagging strategy that enables LC-MS detection of clickable chemoproteomics-modified peptides.

## Methods

### Reagents

Bovine serum albumin (BSA: 99%), dl-dithiothreitol (DTT: 98%), tris((1-benzyl-4-triazolyl)methyl)amine (TBTA: 97%), and copper(II) sulfate (99%) were purchased from Sigma-Aldrich. Iodoacetamide alkyne (98%) was purchased from Click Chemistry Tools, MS grade trypsin protease was purchased from Thermo Scientific, BDP FL azide (BODIPY azide: 99%) was purchased from Lumiprobe, tris(2-carboxyethyl)phosphine HCl (TCEP-HCl: 98%) was purchased from Gold Biotechnology, formic acid (99%) was purchased from Millipore, LC-MS Optima grade water and acetonitrile were purchased from Fisher Chemical and a MassPREP *E. coli* digest standard was purchased from Waters.

### Preparation of a BODIPY-Labeled Peptide Mixture

Bovine serum albumin was reduced with 5 mM DTT at 56 °C for 30 min, alkylated in the dark with 20 mM iodoacetamide alkyne at room temperature for 20 min, and digested with trypsin at a 1:20 enzyme to substrate ratio at 37 °C for 18 h. The alkylated peptides were coupled to BODIPY azide at 37 °C for 18 h using a mixed catalyst of 0.23 mM TBTA, 2.3 mM TCEP-HCl and 2.3 mM copper sulfate (molar ratio of 1:1:2:20:20 BODIPY azide/alkyne/TBTA/TCEP/copper sulfate). Peptides were twice desalted with C18 stage tips, dried using a speedvac, and reconstituted in 0.1% formic acid.

### LC-MS Analysis

Peptides were analyzed using reverse-phase nanoflow liquid chromatography (Thermo Fisher Easy nLC 1200) coupled to an Orbitrap tribrid Fusion Lumos mass spectrometer (Thermo Fisher). The separation was performed on an Ion Opticks 25 cm × 75 um ID, 1.7 um C18 Aurora Ultimate nanoflow packed column. Mobile phase A (MPA) consisted of 0.1% formic acid in water and mobile phase B (MPB) consisted of 0.1% formic acid in 80% acetonitrile. Two different gradients were used for the analyses of BSA and the *E. coli* proteome. For BSA samples, MPB increased from 3% to 30% over 15 min and from 30% to 100% over 15 min, then was held at 100% for 10 min. For the *E. coli* samples, MPB increased from 1% to 15% over 25 min, 15% to 40% over 15 min, 40% to 90% over 5 min, then was held at 90% for 2 min.

The nanoelectrospray ionization (nESI) source was operated at a constant 1900 V in positive ion mode and the %RF was varied from 10 to 60%. DDA experiments were performed with a 2 s cycle between full scans. MS1 spectra were collected from 400 to 1800 *m*/*z* at 50K resolving power with AGC of 7.5E5. Peptides were isolated in the quadrupole using an isolation window of 1.6 *m*/*z* units and subjected to normalized collision energy (NCE) of 30% in the higher-energy collision induced dissociation (HCD) cell followed by analysis in the Orbitrap at 15K resolving power and AGC of 1.0 E5 with 3 microscans. For targeted LC-MS/MS, the instrument cycled through 10 MS2 scans between each MS1 scan. The MS1 scan range was narrowed according to the target precursor ion masses and dynamic exclusion was applied for 1 s. Orbitrap MS2 spectra were acquired at 15K resolving power using HCD with 30% NCE, an isolation window of 1.6, AGC of 5.0 E5, and 3 microscans.

### Peptide Sequencing with Proteome Discoverer

DDA and targeted MS2 data were searched with Proteome Discoverer (Thermo Fisher, version 3.0) using the SEQUEST algorithm. Precursor mass accuracy was set to 10 ppm, peptides were allowed up to two missed cleavages, and peptides were filtered for high confidence in the consensus step. Three custom cysteine variable modifications were applied, corresponding to modifications introduced by iodoacetamide alkyne (C_5_H_5_NO), iodoacetamide alkyne coupled to BDP FL azide (C_22_H_26_BF_2_N_7_O_2_) and iodoacetamide alkyne coupled to BDP FL azide with HF neutral loss (C_22_H_25_BFN_7_O_2_). The exact masses of the BDP FL variable modifications were calculated for the M+1 isotope rather than the monoisotopic mass. Using the ^11^B-containing M+1 isotope ensures matching to the ^11^B precursor ions selected in DDA and for targeted MS2. HF neutral loss was also indicated in the two BDP FL variable modifications to enable matching between BDP FL precursor ions and corresponding b and y fragment ions bearing BDP FL (−HF) or (−2HF) neutral loss.

## Results and Discussion

### Peptide Isotopic Distributions with Boron

**Figure 1:**
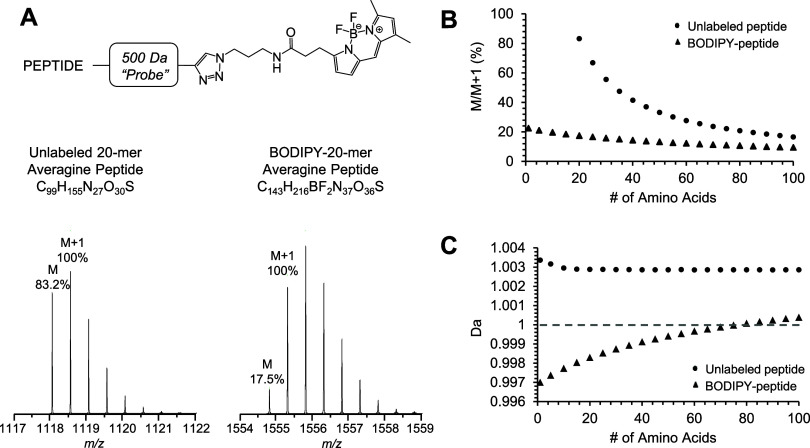
(A) Chemical structure of BODIPY clicked to a theoretical 500 Da probe coupled to a tryptic peptide, and simulated doubly charged isotopic distributions of theoretical unlabeled (C_99_H_155_N_27_O_30_S) and BODIPY-labeled (C_143_H_216_BF_2_N_37_O_36_S) peptides with 20 averagine amino acids. (B) Percent relative intensity of the M to M+1 peak for simulated BODIPY- versus unlabeled peptides (Note: M/M+1 (%) data that exceeds 100% not shown for unlabeled peptides <20 amino acids). (C) Exact mass difference between the M and M+1 peak for BODIPY- versus unlabeled peptides.

The incorporation of bromine to chemoproteomics labels has proven effective at differentiating tagged versus untagged tryptic peptides.^29−32^ However, the dibromine-containing isotopic distribution is complex requiring a deconvolution algorithm for scoring. The theoretical figures of merit for boron-containing chemoproteomics labels should in principle provide a more straightforward discrimination. To explore the theoretical figures of merit and applicability in chemoproteomics, we assessed the theoretical search space for differentiating BODIPY-labeled peptides from unlabeled peptides based on two analytical figures of merit: (1) isotopic distribution abundances and (2) exact mass difference. Isotopic distributions were simulated in Freestyle for averagine^40^ peptides modified with a generic small molecule probe (C_27_H_40_N_4_O_5_, MW = 500 Da) and conjugated to BODIPY azide (C_17_H_21_BF_2_N_6_O), as shown in Figure 1A. For the generic probe, we are assuming a molecular weight close to small molecule drugs and assume it is only comprised of carbon, hydrogen, nitrogen, and oxygen. Additional atoms found in small molecule drugs such as sulfur, chlorine and bromine would in theory alter the isotopic distribution to varying degrees. Representative simulated isotopic distributions for unlabeled and BODIPY-labeled 20-mer peptides are shown in Figure 1A. The ^10^B monoisotopic peak (M) relative to the M+1 peak clearly distinguishes the BODIPY peptide from its unlabeled analogue.

Isotopic distributions were simulated for the BODIPY-labeled peptides and analogous unlabeled peptides up to 100 amino acids in length (>10000 Da). The M to M+1 peak intensity ratios for BODIPY- versus unlabeled peptides are plotted in Figure 1B. The number of amino acids plotted far exceeds the practical tryptic peptide size of 7–35 amino acids in the human proteome^41^ assuming up to two missed cleavages. The M to M+1 relative intensity for BODIPY-labeled peptides within the practical range was 20.5–15.1%, compared with 242.7–47.5% for unlabeled peptides. The notable flatness of the curve of M to M+1 peak intensity for BODIPY-labeled peptides suggests a narrow and specific window for computational detection of the boron isotopes. We speculate that boron may afford detection with greater specificity compared with the dibromide motif because the heavy isotope ^11^B is concentrated in the M+1 peak which is more isotopically pure than the M + 2 peak enhanced by ^81^Br. A comparative isotopic simulation of averagine peptides modified with a single boron atom versus two bromine atoms is presented in Supporting Information, Figure S2.

The mass defect of an isotope is defined as the difference between its nominal mass and exact mass, with ^12^C as the standard with zero mass defect (12.00000 Da).^42^ The large and negative mass defect of ^79^Br (−0.08166 Da) has been used in intact protein analysis^43^ and peptide labeling,^44^ by shifting the monoisotopic mass into unique zones of the mass spectrum. Further analysis of the BODIPY-labeled peptides reveals a shift to higher monoisotopic mass due to the positive mass defect of ^10^B (+0.01294 Da),^37^ which is measurable by calculating the difference in exact mass between M and M+1. The exact mass difference between M and M+1 plotted in Figure 1C was 0.99753–0.99893 Da for BODIPY-labeled peptides compared with 1.00303–1.00287 Da for unlabeled peptides over the 7–35 amino acid tryptic peptide range. This affords a second level of discrimination of BODIPY-labeled peptides. Since both analytical figures of merit (intensity ratio, mass defect) are based on the relationship between the M and M+1 peaks in the peptide isotopic distribution, the development and application of computational tools to search for boron encoded peptides should be relatively straightforward.

### Characterization of Signature HF Neutral Loss

**Figure 2:**
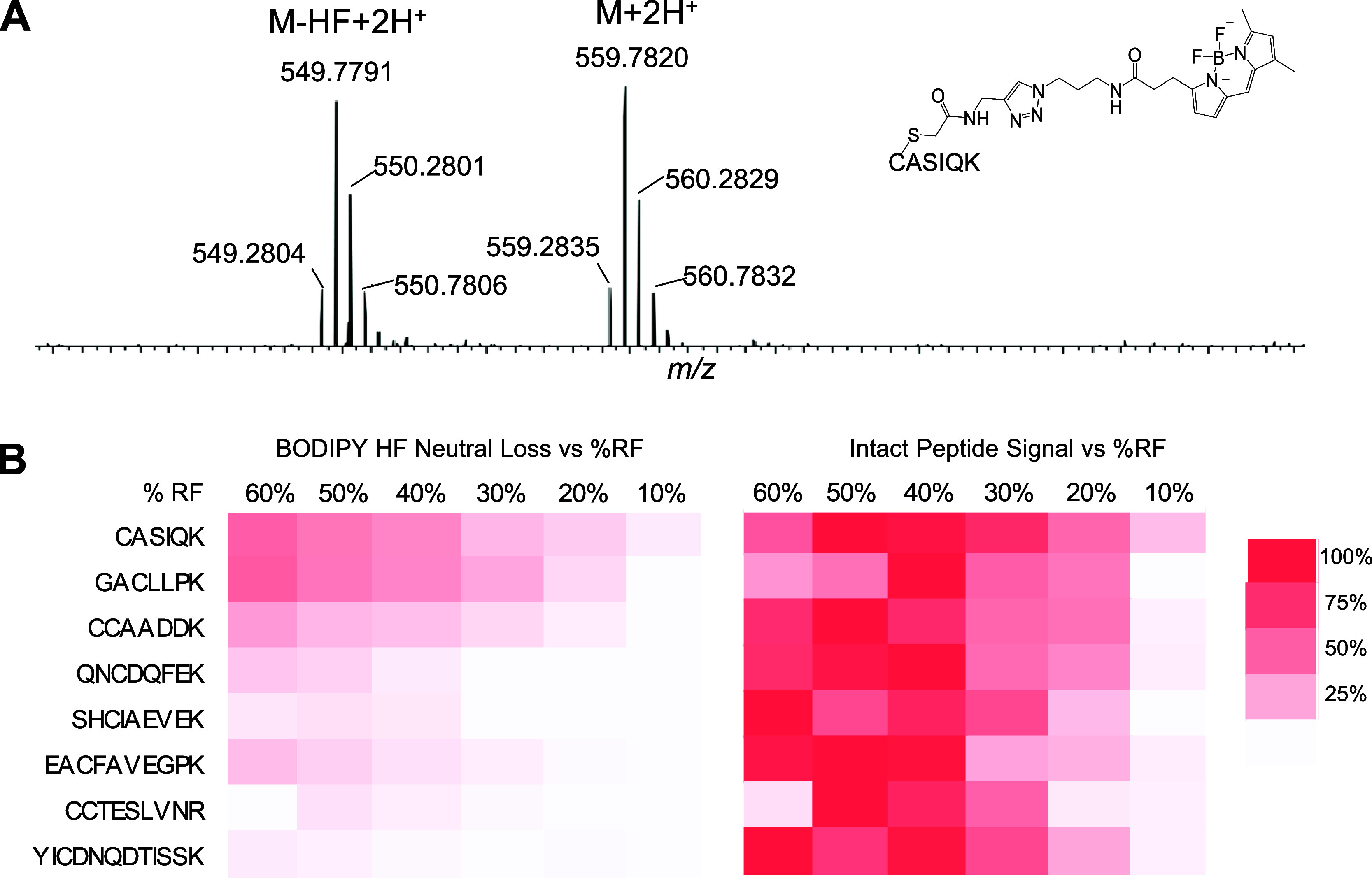
(A) MS1 spectrum of representative BODIPY peptide C*ASIQK demonstrates significant HF neutral loss. (B) The effect of decreasing % RF on BODIPY HF neutral loss and absolute intact peptide signal in MS1 spectra.

A BSA digest containing BODIPY-labeled peptides was analyzed using DDA. LC-MS/MS analyses revealed the presence of prominent precursor ion pairs separated by lower *m*/*z* species with a mass difference of −20.006 Da. Lastly, the introduction of the BODIPY tag increased the average elution time by ∼7 min (Figure S3) relative to their unlabeled peptide counterparts and did not affect peptide identification. Further analysis attributed this to neutral loss from in-source fragmentation of hydrofluoric acid (HF) which was previously reported for the collision induced dissociation of BODIPY analogues.^45^ A representative mass spectrum of the BODIPY-tagged peptide C*ASIQK (Figure 2A) exhibited a ∼50% split signal due to the neutral loss of HF. It was observed that some peptides also exhibited loss of 2HF in MS1 spectra, but these species were much less prominent compared with the loss of a single HF fragment. In an effort to characterize the interplay between HF neutral loss, precursor abundance, and ESI source RF lens voltage, we systematically varied the lens voltage (%RF) from 10 to 60% by 10% increments and monitored eight representative BODIPY-tagged peptides for single HF neutral loss and absolute signal (Figure 2B). HF neutral loss decreased with decreasing %RF, but the extent of neutral loss ranged significantly among the different peptides. Maximum absolute signal for each of the eight peptides was achieved between 60 and 40% RF.

**Figure 3:**
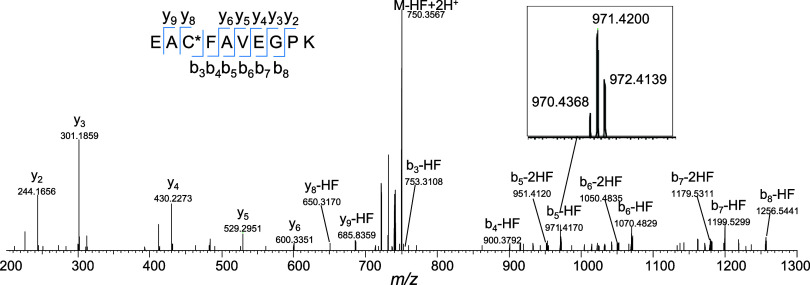
MS2 spectrum of BODIPY-labeled peptide EAC*FAVEGPK at HCD 30 shows HF and 2HF neutral loss and retention of the ^10^B peak in fragment ions.

BODIPY-labeled peptides can also undergo HF and 2HF neutral losses during HCD MS/MS experiments. A representative HCD mass spectrum of BODIPY-tagged EAC*FAVEGPK at 30% NCE is shown in Figure 3, where b and y ions that retained the modified cysteine showed the signature HF neutral loss and signature ^10^B peak, which enabled the localization of the amino acid labeling site. Furthermore, MS/MS of BODIPY (-HF) precursors (ie. first HF loss occurred in MS1) also show a loss of the second HF during fragmentation (*vide infra*). In order for peptide sequencing software to bridge the gap in exact mass between precursor ions and corresponding b and y fragment ions that have lost HF in the MS/MS experiment, a search engine with a user-defined neutral loss parameter must be used, otherwise the identification can be missed. Using Proteome Discoverer, HF neutral loss was specified in the chemical modification editor individually for the relevant variable modifications, and data was searched using the SEQUEST algorithm. For example, searching without HF neutral loss in the BODIPY variable modification parameter did not identify the spectrum in Figure 3, however, by editing the variable modification to consider HF neutral loss, SEQUEST was able to detect the correct sequence.

### Identifying Boron Encoded Peptides in Complex Mixtures

**Figure 4:**
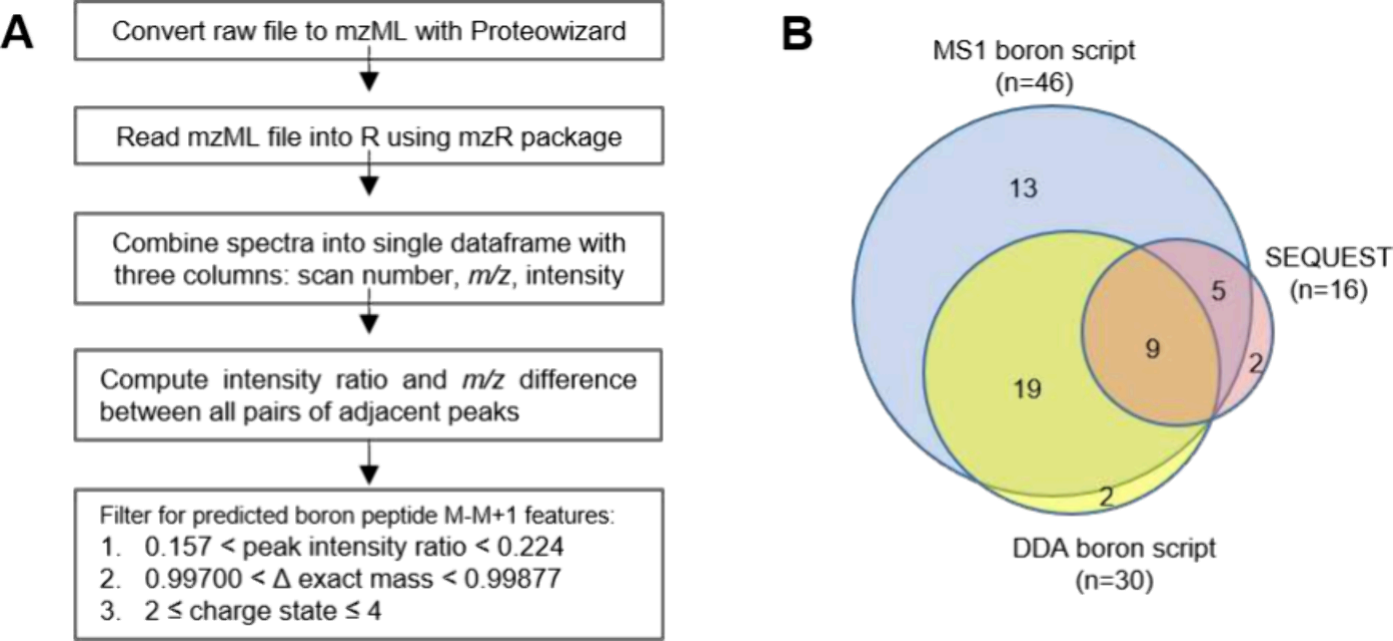
(A) Workflow summary for the computational detection of boron encoded peptides (B) BODIPY peptide coverage achieved with MS1 and data dependent acquisition (DDA) searched with the boron script vs SEQUEST.

An R script was developed to detect boron encoded peptides in complex LC-MS data sets (code provided in Supporting Information, Figure S1). An overview of the raw data processing workflow is summarized in Figure 4A. First a raw MS file is centroided and converted to mzML format using ProteoWizard msConvert,^46^ and the mzML file is read into R using functions from the “mzR” package.^47^ Peaks from all mass spectra are combined into a single dataframe, with each observation containing the original scan number, *m*/*z* value and absolute intensity. The script then computes the intensity of each peak relative to the peak that immediately follows, as well as the difference in *m*/*z* between all adjacent peaks. Three filters are then applied to select for the (M, M+1) pairs of boron encoded peptides: intensity ratio, exact mass difference and charge state. In this work, the predicted ranges in intensity ratio (0.157–0.224) and exact mass difference (0.99700–0.99877 Da) between the M and M+1 peaks were informed by simulated averagine peptide isotopic distributions of 5–35 amino acids modified with C_22_H_26_BF_2_N_7_O_2_ (acetamide alkyne–azide BODIPY). The script parameters can be modified for chemoproteomics probes with different chemical formulas that differ in size or contain additional atomic species.

To compare the BODIPY peptide coverage achieved using computational boron pattern detection in R versus the traditional DDA-peptide sequencing approach, we first generated a fixed list of theoretical BSA BODIPY-labeled peptide precursor ions to serve as a reference database. *In silico* tryptic digest of BSA was performed with ExPasy peptide cutter, returning 25 cysteine-containing peptides of 6–22 amino acids. Exact mass of the acetamide alkyne–azide BODIPY modification was added to the exact mass of each peptide, and theoretical *m*/*z* values were tabulated for charge states 2 through 4. The list was then duplicated to include HF neutral loss. For peptides containing more than one cysteine, only one position was conjugated to BODIPY, and the other position(s) were merely alkylated.

The BSA sample was analyzed under both DDA and continuous MS1 conditions, and both files were processed through the R script to search for the boron encoded peptides. The DDA file was also searched using SEQUEST. Precursor ions found using R and SEQUEST were cross searched against the reference database of theoretical BODIPY peptide precursor ions generated from *in silico* digestion of BSA. Peptide coverage achieved using all three search strategies is summarized in Figure 4B. A total of 46 BODIPY precursor ions were found by searching the continuous MS1 data set for boron encoded peptides, corresponding to 22 out of the possible 25 cysteine-containing BSA peptides. Comparatively, 30 precursors were found by searching the DDA file for boron encoded peptides and only 16 precursors were found by searching the DDA file with SEQUEST. The additional 13 precursor ions found by the script in the continuous MS1 experiment versus DDA demonstrates how DDA can limit identification of low abundant peptides simply because the shorter elution window may elapse while the instrument is busy acquiring higher abundance-triggered scans. Targeted profiling approaches that rely on isotopic encoding can help to circumvent the abundance bias because they offer more systematic precursor screening compared with DDA.

To estimate a false positive rate for the boron detection script, an *E. coli* digest standard assumed to not contain any endogenous boron was analyzed with continuous MS1 scanning and data was processed through the R script. 2403 hits were returned in the script output from a data set of 24.5 million peaks, corresponding to a false positive rate (FPR) of 0.01%. We attribute the low false positive rate to the three stages of filtering in the script: intensity ratio, mass defect and charge state. The script was modified to exclude the mass defect and charge state filters and the FPR was recalculated (Table 1). First we widened the mass difference parameter to 0.99700–1.00316 Da such that it may include the mass difference observed in unlabeled averagine peptides, effectively removing the ^10^B mass defect filter, and the FPR tripled to 0.03%. We then entirely eliminated the exact mass difference and charge state filters to evaluate the false positive rate of a script based on isotopic peak intensities alone, and the false positive rate increased markedly to 2.9%, indicating a loss of specificity.

**Table 1:** Number of False Positives and False Positive Rate (FPR) Returned for the Boron R Script with Decreasing Levels of Peak Filtering

M-M+1 peak filters	false positives	FPR (%)
intensity, mass defect, charge state	2403	0.01
intensity, charge state	7426	0.03
intensity	705, 102	2.90

### Targeted Profiling of BODIPY Peptides

**Figure 5:**
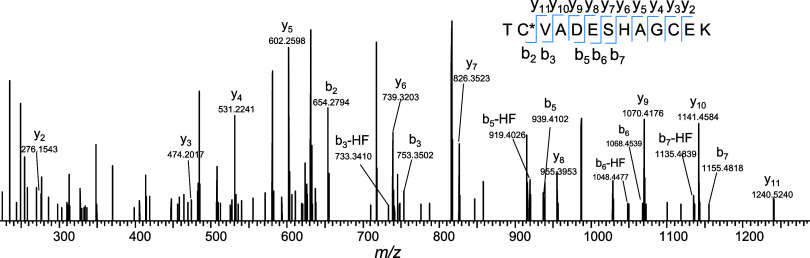
MS2 spectrum of BODIPY-labeled peptide TC*VADESHAGCEK identified using targeted profiling.

We next evaluated the BODIPY labeling strategy for targeted profiling within a complex proteomic background. The BSA BODIPY peptide mixture was spiked into an *E. coli* digest standard and analyzed with DDA and SEQUEST. Only one BODIPY-labeled peptide was found. The sample was then reanalyzed with continuous MS1 scanning and searched with the R script for boron encoded peptides and the script returned 2764 hits. The hits from the script output were grouped by unique *m*/*z* values, and the list was refined to remove presumed false positives and noisy peaks. Hits were removed which were either detected outside of the peptide elution window, found in only a single spectrum, or found across several chromatographically unrelated spectra. The remaining 273 *m*/*z* values were appended with retention times to generate an inclusion list for targeted MS2 experiments. The targeted MS2 data was searched with SEQUEST and three additional BODIPY-labeled peptides were found. A representative MS2 spectrum is shown in Figure 5 for BODIPY peptide TC*VADESHAGCEK which was identified using the targeted approach. The BODIPY tag conjugated to the precursor ion in Figure 5 had lost the signature HF fragment in the ESI source, and the b-ion series demonstrated loss of the second HF fragment in the MS/MS experiment.

## Conclusions

The detection and identification of low abundant, nontemplate modified peptides in shotgun proteomics poses many technical challenges and one solution is to decouple the discovery of labeled peptides from the sequencing step. Isotopic encoding with bromine, chlorine, and selenium for targeted profiling have demonstrated improved peptide coverage because the isotopic signatures are detectable in the peptide MS1 spectrum for systematic precursor screening. In this work, we investigated boron-containing BODIPY as an alternative, commercially available, chemoproteomic labeling reagent.

Boron imparts an isotopic signature between the monoisotopic and M+1 peak which was computationally identifiable using a newly developed R script. The script incorporates three stages of peak filtering (intensity ratio, mass defect, charge state) resulting in a low false positive rate (0.01%). Processing an MS1 dataset of BODIPY cysteine-modified BSA with the R script resulted in three times the peptide coverage achieved with DDA and peptide sequencing software. The BSA BODIPY sample spiked into the *E. coli* proteome and analyzed with targeted profiling resulted in the detection of four labeled peptides compared with one peptide detected with standard DDA.

The BODIPY labeling strategy can be applied to any chemoproteomic investigation where the probe contains a clickable handle and where the site(s) of labeling are heterogeneous or unknown. Future development work could be done to incorporate the boron detection R script into a node within commercial sequencing software, or even be applied in real time to trigger targeted MS2 experiments of boron encoded peptides. While this work focuses on the mass spectrometric properties of BODIPY (e.g., isotopic signature, gas-phase fragmentation), the use of fluorescence or UV spectroscopy is inherently feasible as previously demonstrated by Yang et al. using dibromine coumarin^32^ or as part of an in-gel digestion workflow.

## Supporting Information Available

Figure S1: R script code for filtering BODIPY-labeled peptides from complex LC-MS data sets. Figure S2: Isotopic simulations comparing boron- and bromine-containing 20-mer averagine peptides. Figure S3: Retention time shifts for acetamide- versus BODIPY-modified BSA peptides (PDF)
