# Supplementary material for: Investigation and Development of the BODIPY-Embedded Isotopic Signature for Chemoproteomics Labeling and Targeted Profiling
Source: J Am Soc Mass Spectrom. 2024 Sep 16;35(10):2440–7. doi: 10.1021/jasms.4c00246 (PMC11457305; doi:10.1021/jasms.4c00246)
Supplement: Supplementary file 1 — js4c00246_si_001.pdf [file js4c00246_si_001.pdf]

Supplementary Material for:

Investigation and Development of the BODIPY-embedded Isotopic Signature for Chemoproteomics Labeling and Targeted Profiling

Rachel Joshi[1] and Adam M. Hawkridge[2]

Departments of Medicinal Chemistry[1] and Pharmaceutics[2]
Virginia Commonwealth University, Richmond, Virginia

Corresponding Author:
Adam M. Hawkridge

```
my_file <- “BODIPY_DATASET.mzML"

BiocManager::install("mzR", force = TRUE)
library("mzR")
library("tidyverse")
rw <- openMSfile(my_file)
spl <- spectra(rw)
number_of_spectra <- length(spl)
scan_number <- 1:number_of_spectra
spl_map2 <- map2(spl, scan_number, ~cbind(.x, scan_number = .y))
raw_data <- do.call(rbind.data.frame, spl_map2)

bodipy_hits <- raw_data %>%
 mutate(mz_B11_peak = lead(mz, default = first(mz))) %>%
 mutate(intensity_B11_peak = lead(intensity, default = first(intensity))) %>%
 mutate(intensity_ratio = intensity / intensity_B11_peak) %>%
 mutate(mz_difference = mz_B11_peak - mz) %>%
 filter((intensity_ratio > 0.157 & intensity_ratio < 0.224) & ((mz_difference > 0.49850 &
mz_difference < 0.49939) | (mz_difference > 0.33233 & mz_difference <  0.33292)|
(mz_difference > 0.24925 & mz_difference < 0.24969))) %>%
 mutate(mz_difference_truncated = trunc(mz_difference*10^2)/10^2) %>%
 mutate(charge_state = ifelse(mz_difference_truncated == 0.49, 2,
ifelse(mz_difference_truncated == 0.33, 3, 4))) %>%
 mutate(mz_B10_peak = mz) %>%
 mutate(intensity_B10_peak = intensity) %>%
 select(scan_number, mz_B10_peak, mz_B11_peak, charge_state, intensity_B10_peak,
intensity_B11_peak) %>%
 arrange(mz_B10_peak)

bodipy_hits
```

Figure S1. R script for filtering BODIPY-labeled tryptic peptides from a centroided mzML file

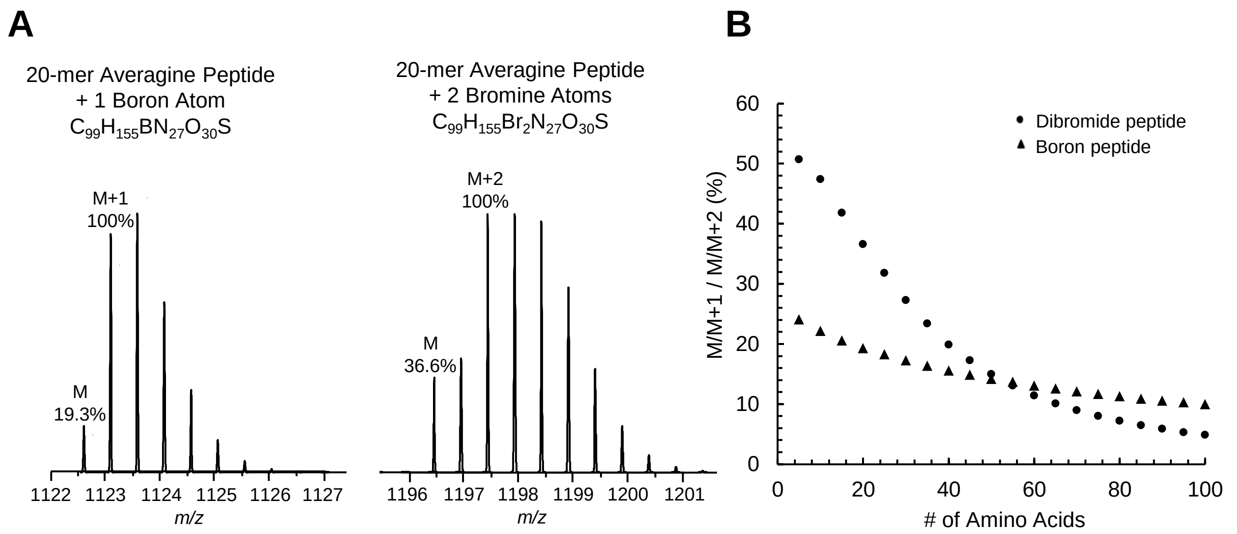

Figure S2. (A) Simulated doubly charged isotopic distributions of theoretical peptides of 20 averagine amino acids ($C_{99}H_{155}N_{27}O_{30}S$) plus one boron atom versus two bromine atoms (B) Percent relative intensity of the M to M+1 peak of the boron-containing averagine peptides versus percent relative intensity of the M to M+2 peak of the dibromine-containing averagine peptides

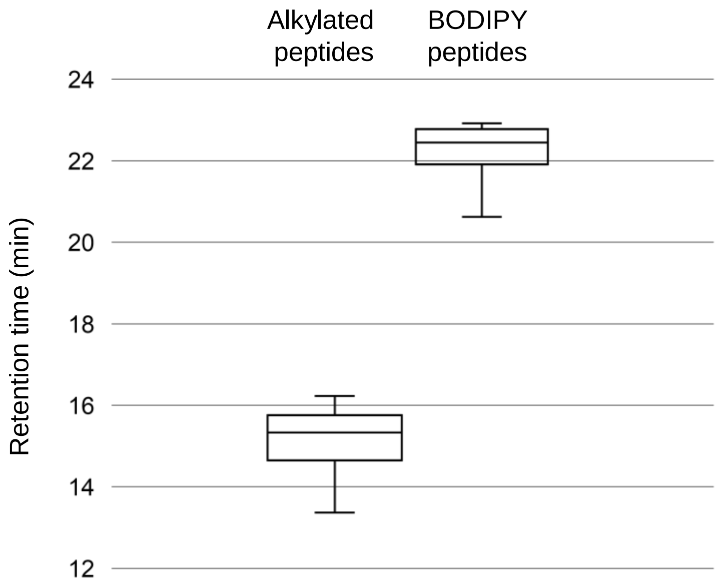

Figure S3. Retention times of ten representative BSA peptides with acetamide alkyne versus acetamide alkyne-azide BODIPY cysteine modifications
